# Live attenuated influenza A virus vaccines with modified NS1 proteins for veterinary use

**DOI:** 10.3389/fcimb.2022.954811

**Published:** 2022-07-22

**Authors:** Aitor Nogales, Marta L. DeDiego, Luis Martínez-Sobrido

**Affiliations:** ^1^ Centro de Investigación en Sanidad Animal (CISA), Centro Nacional Instituto de Investigación y Tecnología Agraria y Alimentaria (INIA, CSIC), Madrid, Spain; ^2^ Department of Molecular and Cell Biology, Centro Nacional de Biotecnología (CNB-CSIC), Campus Universidad Autónoma de Madrid, Madrid, Spain; ^3^ Department of Disease Intervention and Prevetion, Texas Biomedical Research Institute, San Antonio, TX, United States

**Keywords:** influenza A virus, non-structural 1 (NS1) protein, live-attenuated influenza vaccine (LAIV), differentiating infected from vaccinated animals (DIVA), interferon

## Abstract

Influenza A viruses (IAV) spread rapidly and can infect a broad range of avian or mammalian species, having a tremendous impact in human and animal health and the global economy. IAV have evolved to develop efficient mechanisms to counteract innate immune responses, the first host mechanism that restricts IAV infection and replication. One key player in this fight against host-induced innate immune responses is the IAV non-structural 1 (NS1) protein that modulates antiviral responses and virus pathogenicity during infection. In the last decades, the implementation of reverse genetics approaches has allowed to modify the viral genome to design recombinant IAV, providing researchers a powerful platform to develop effective vaccine strategies. Among them, different levels of truncation or deletion of the NS1 protein of multiple IAV strains has resulted in attenuated viruses able to induce robust innate and adaptive immune responses, and high levels of protection against wild-type (WT) forms of IAV in multiple animal species and humans. Moreover, this strategy allows the development of novel assays to distinguish between vaccinated and/or infected animals, also known as Differentiating Infected from Vaccinated Animals (DIVA) strategy. In this review, we briefly discuss the potential of NS1 deficient or truncated IAV as safe, immunogenic and protective live-attenuated influenza vaccines (LAIV) to prevent disease caused by this important animal and human pathogen.

## Introduction

### Influenza A virus

**Figure 1:**
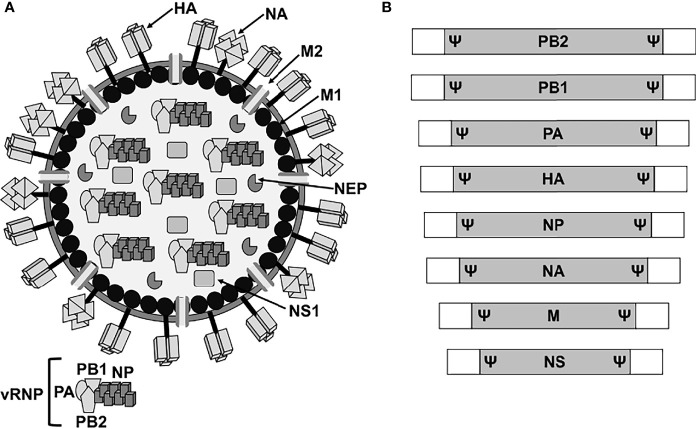
IAV virion structure and genome organization. **(A)** Virion structure. IAV particles have a lipid envelope where the two major viral glycoproteins HA and NA and the ion channel M2 are located. Below the viral lipid membrane is a layer composed of M1 protein and the NEP. Inside the viral particle are the vRNP particles formed by the vRNA coated by the viral NP and linked to the heterotrimeric polymerase complex (PB2, PB1 and PA). **(B)** Genome organization. IAV contains eight vRNA segments (PB2, PB1, PA, HA, NP, NA, M, and NS) made of a coding region (gray boxes) flanked at the 3′ and 5′ terminal ends by untranslated non-coding regions (white boxes). At the end of the 3′ and 5′ coding regions are the specific viral segment packaging signals (Ψ) required for efficient encapsidation of the vRNP particles into new viruses.

Influenza A viruses (IAV) belong to the *Orthomyxoviridae* family, which contains a lipid envelope enclosing the viral genome formed by eight negative sense, single-stranded, RNA segments (Shaw, 2007). The viral RNA (vRNA) segments contain a long central coding region flanked at 3′ and 5′ termini by non-coding regions (NCR), which work as promoters for viral replication and transcription (Shaw, 2007). In addition, the 3′ and 5′ end of the coding regions contain the packaging signals (Ψ) for the efficient encapsidation of the viral genome (Shaw, 2007; Boivin et al., 2010; Baker et al., 2014; Gerber et al., 2014; Pohl et al., 2016; Martinez-Sobrido et al., 2018; Fan et al., 2019) (**Figure 1**). The eight vRNAs encode for the three components of the viral polymerase complex, the polymerase basic 2 and 1 (PB2 and PB1, respectively) and acidic (PA) proteins, the two surface glycoproteins hemagglutinin and neuraminidase (HA and NA, respectively), the nucleoprotein (NP), the matrix protein 1 (M1), the membrane protein 2 (M2), the non-structural (NS1) protein, and the nuclear export protein (NEP) (**Figure 1**). The IAV genome also encodes for other viral proteins through multiple mechanisms (Shaw, 2007; Gamblin and Skehel, 2010; Hai et al., 2010; Bavagnoli et al., 2015; Nogales et al., 2018b). Each of the vRNAs are arranged as viral ribonucleoprotein complexes (vRNPs), where vRNAs are coated with multiple subunits of the viral NP and associated with one copy of the heterotrimeric polymerase complex formed by one copy of the PB2, PB1, and PA proteins (Shaw, 2007; Boivin et al., 2010; Pohl et al., 2016; Martinez-Sobrido et al., 2018; Fan et al., 2019) (**Figure 1**). IAV are subtyped based on the genetic and antigenic properties of the viral HA and NA glycoproteins, which are also the main target of neutralizing antibodies induced after vaccination and/or natural viral infection (Shaw, 2007; Parrish et al., 2015). HA is responsible for the attachment of IAV to target cells for viral entry, while NA facilitates egress from virus-infected cells (Gamblin and Skehel, 2010; McAuley et al., 2019; Wille and Holmes, 2019; de Vries et al., 2020; Wu and Wilson, 2020; Sempere Borau and Stertz, 2021).

IAV are among the most challenging pathogens, causing a great impact in human and animal health (Cox et al., 2009; Dorratoltaj et al., 2017; Mancera Gracia et al., 2020; Oladunni et al., 2021; Salvesen and Whitelaw, 2021). IAV are able to infect multiple animal species, including waterfowl, poultry, swine, horses, dogs, cats, bats, multiple marine mammals as whales or seals, and humans. Waterfowl of the orders *Anseriformes* (ducks) and *Charadriiformes* (shorebirds, gulls) have been considered the most important reservoir hosts reaching prevalence levels of >20% in the migration season (Shaw, 2007; Latorre-Margalef et al., 2014; Parrish et al., 2015; Short et al., 2015; Sutton, 2018; Wille and Holmes, 2019). These waterbirds harbor 16 HA and 9 NA genes subtypes, and usually experience clinically asymptomatic infections. Importantly, since more sequencing efforts have been implemented during the last years, mammals, such as swine, are emerging as key IAV reservoirs (Parrish et al., 2015; McLean and Graham, 2022). Recently, the phylogenetic diversity of IAV has increased with the identification of H17N10 and H18N11 subtypes of IAV in fruit bats (Zhu et al., 2012; Zhu et al., 2013; Yang et al., 2021).

Given that the IAV genome contains eight vRNA segments, and that several IAV strains can infect the same host, viral genomic reassortment, or antigenic shift, occurs frequently (Marshall et al., 2013; Steel and Lowen, 2014; Lowen, 2017; Lowen, 2018; White et al., 2019). As a consequence of reassortment, viral progeny can harbor new genomic constellations from the different original viruses. In fact, this process is highly important to understand IAV evolution, ecology, and adaptation to new hosts, because new viruses can, among others, obtain advantages in viral replication, transmission, or the ability to evade host immune responses (Parrish et al., 2015; Lowen, 2017; Lowen, 2018; Wille and Holmes, 2019). In addition, antigenic drift has been described to be responsible for the emergence of novel IAV variants, when mutations in the viral genome are introduced and selected in the viral population (Shaw, 2007; Parrish et al., 2015; Short et al., 2015; Sutton, 2018; Wille and Holmes, 2019; McLean and Graham, 2022).

The continuous adaptation of IAV to new host species represents an important concern, and multiple studies focused on understanding the evolutionary pressures that drive IAV host adaptation and transmission are being carried out (Imai and Kawaoka, 2012; Short et al., 2015; Johnson and Ghedin, 2019). Moreover, IAV diversity and complexity highlight the relevance of the one-health approach to study IAV infections and spread, with the goal to reduce the impact of influenza disease on healthcare systems, animal welfare or food production (e.g., poultry industry) (Cox et al., 2009; Dorratoltaj et al., 2017; Mancera Gracia et al., 2020; Oladunni et al., 2021; Salvesen and Whitelaw, 2021). In addition, due to IAV infections across so many different animal species, there are continuous and transient spillover infections, which occasionally can result in sustained epidemic transmission (Short et al., 2015; Wille and Holmes, 2019; Wang et al., 2021; McLean and Graham, 2022). Stable transmission in new hosts depends on IAV and host ecology as well as the acquisition of specific genetic changes in the viral genome that allow productive infections. Because of that, most of IAV host-jumping events are not successful (Shaw, 2007; Parrish et al., 2015; Short et al., 2015; Rajao et al., 2018; Sutton, 2018; Wille and Holmes, 2019; Oladunni et al., 2021; Verhagen et al., 2021; McLean and Graham, 2022; Murakami et al., 2022).

### Reverse genetics techniques for the development of IAV vaccines based on modifications in the NS1 protein

Reverse genetics methods have provided researchers with a powerful experimental approach to generate recombinant IAV from cloned complementary (c)DNAs (Luytjes et al., 1989) (Hoffmann et al., 2000; Nogales and Martinez-Sobrido, 2016; Nogales et al., 2017d; Blanco-Lobo et al., 2019). These reverse genetics techniques have allowed investigators to study basic aspects of the biology of IAV, including, among others, the identification of host factors that control viral cell entry, genome viral replication and transcription, and viral assembly and budding (Rossman and Lamb, 2011; Chua et al., 2013; Baker et al., 2014; Khaperskyy et al., 2016). Moreover, these reverse genetics systems have been used to rescue recombinant IAV with predetermined mutations in their viral genomes to examine their contribution to viral replication, pathogenesis, and transmission; and to generate attenuated forms for their use as live-attenuated influenza vaccines (LAIV) for the prevention of IAV infections (Nogales et al., 2016b; Nogales and Martinez-Sobrido, 2016; Clark et al., 2017; Nogales et al., 2017a; Nogales et al., 2017c; Nogales et al., 2017f; Chauche et al., 2018; Nogales et al., 2018a; Rodriguez et al., 2018a; Rodriguez et al., 2018b; Hilimire et al., 2020). Notably, reverse genetics systems have leaded to the implementation of replicating competent or deficient IAV expressing one or two reporter genes (Nogales et al., 2015; Breen et al., 2016a; Breen et al., 2016b; Nogales et al., 2016a; Nogales et al., 2019a). These viruses have been an important biotechnological tool to track viral infections using *in vitro* and *in vivo* imaging systems (Nogales et al., 2015; Breen et al., 2016a; Breen et al., 2016b; Nogales et al., 2016a; Nogales et al., 2019a). Moreover, reporter-expressing IAV have been used to identify antivirals or neutralizing antibodies using high throughput screening (HTS) approaches (Nogales et al., 2015; Breen et al., 2016a; Breen et al., 2016b; Nogales et al., 2016a; Nogales et al., 2019a).

**Figure 2:**
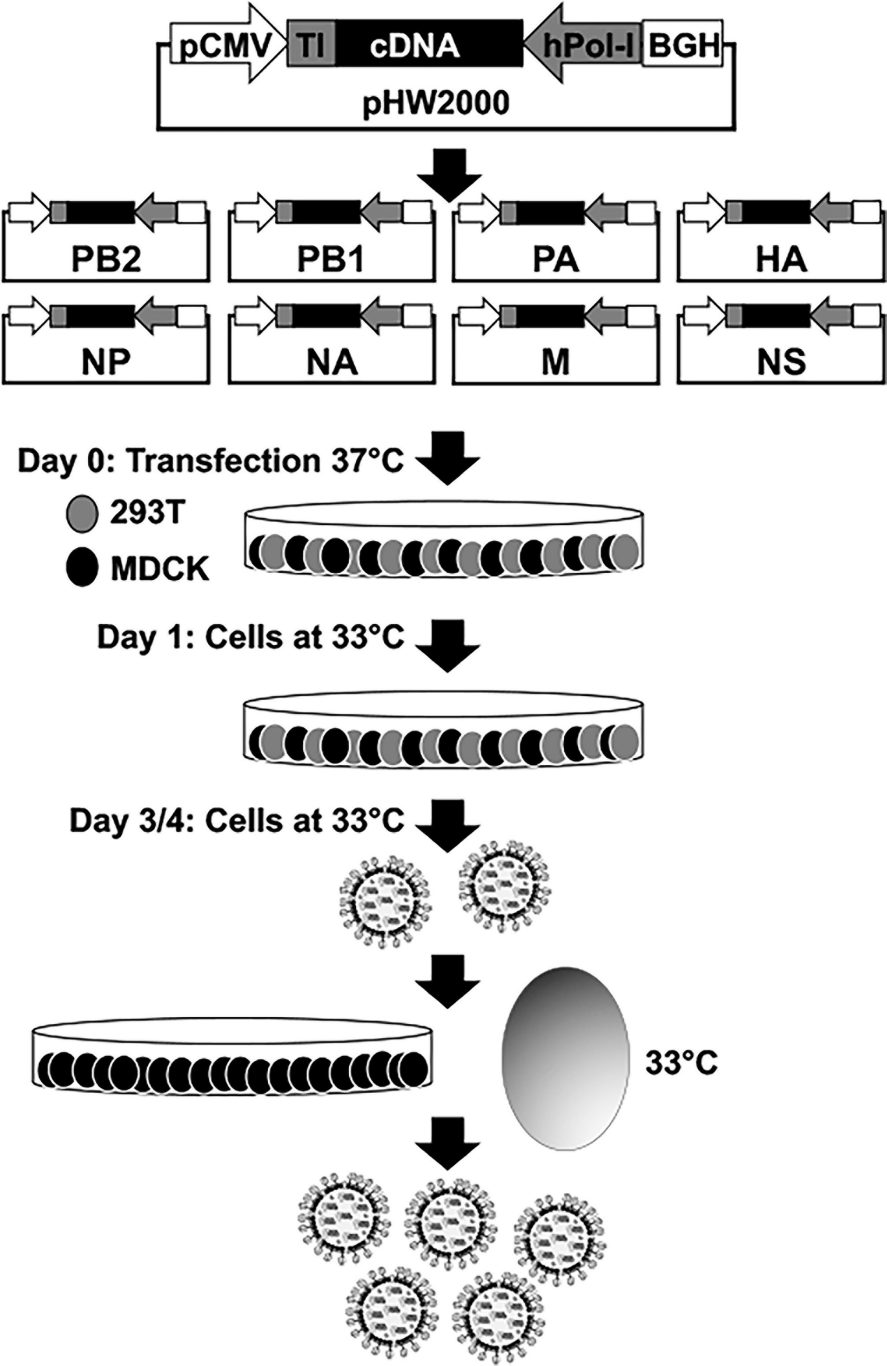
Plasmid-based reverse genetics approach for the recovery of recombinant IAV. Schematic representation of the ambisense plasmid used to generate recombinant IAV is shown in the top. IAV cDNAs are cloned into the plasmid flanked by the human polymerase I promoter (hPol-I, gray arrow) and the mouse Pol-I terminator (TI, gray box) sequences to drive the synthesis of the vRNAs. In opposite orientation to the polymerase I cassette is the polymerase II (Pol-II) cassette made of a Pol-II dependent cytomegalovirus promoter (pCMV, white arrow) and the polyadenylation sequence of the bovine growth hormone (BGH, white box) to allow the expression of viral proteins from the same viral cDNAs. Co-cultures of human 293T (gray) and canine MDCK (black) cells are co-transfected with the eight (PB2, PB1, PA, HA, NP, NA, M, and NS) ambisense plasmids. Recovered virus is amplified in fresh cells or embryonated chicken eggs for vaccine production. Since NS1 truncated or deficient IAV have a *ts* phenotype, virus rescue and amplification is carried out at 33°C.

The most extended plasmid-based reverse genetics approach for the rescue of recombinant IAV is based on the use of eight ambisense plasmids, one per IAV viral segment, which allows for the simultaneous expression of the viral proteins and the vRNAs in susceptible cells (**Figure 2**). Recombinant viruses can be recovered from the tissue culture supernatants of cells co-transfected with the eight plasmids and the recovered virus can be propagated in fresh cells or embryonated chicken eggs (**Figure 2**) (Hoffmann et al., 2000; Martinez-Sobrido and Garcia-Sastre, 2010; Nogales and Martinez-Sobrido, 2016; Nogales et al., 2017d). Importantly, the use of eight-plasmid reverse genetics systems have allowed the easy modifications of each viral gene individually. This advantage has been broadly used to generate LAIV by introducing specific amino acid substitutions, or deletions, in the viral genome (Martinez-Sobrido et al., 2018; Rodriguez et al., 2018b; Blanco-Lobo et al., 2019; Hilimire et al., 2020). For instance, IAV encoding truncated versions of NS1 or where the NS1 sequence was completely removed (ΔNS1) have been generated and used as potential LAIV candidates in multiple animal species (Quinlivan et al., 2005; Solorzano et al., 2005; Richt et al., 2006; Vincent et al., 2007; Wang et al., 2008; Chambers et al., 2009; Steel et al., 2009; Kappes et al., 2012; Choi et al., 2015; Jang et al., 2016; Na et al., 2016; Chen et al., 2017; Nogales et al., 2017b; Jang et al., 2018; Nicolodi et al., 2019; Lee et al., 2021; Vandoorn et al., 2022a), or to study IAV infections (Nogales et al., 2021a), including the contribution of NS1, and its domains, in viral pathogenesis (Nogales et al., 2017a; Nogales et al., 2017c; Chauche et al., 2018; Nogales et al., 2018a; Nogales et al., 2018b; Nogales et al., 2021b). Because NS1 is the main countermeasure against cellular antiviral responses, the recovery of NS1 truncated or deficient viruses can be challenging, since these viruses have limited ability to inhibit the cellular innate immune responses induced during viral infection (Garcia-Sastre A et al., 1998; Kochs et al., 2007; Hale et al., 2008; Nogales et al., 2018b; Nogales et al., 2019b).

### NS1 protein structure and functions

**Figure 3:**
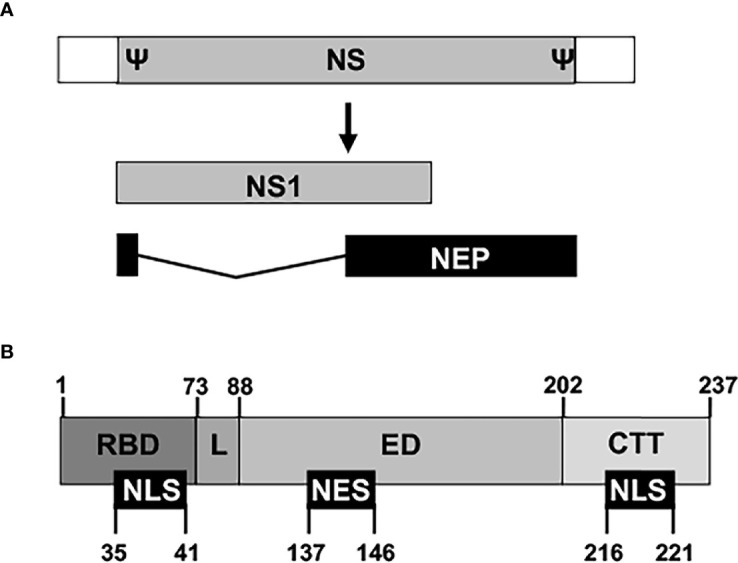
Schematic representation of NS segment and NS1 domains. **(A)** An IAV NS vRNA segment is shown by a gray box and non-coding regions (NCR) are indicated with white boxes. Packaging signals (Ψ) at the end of the 3′ and 5′ coding regions are also indicated. IAV NS1 and NEP transcripts are indicated with gray and black boxes, respectively. IAV NS1 and NEP ORFs share the first 10 amino acids in the N-terminus. **(B)** The NS1 protein is divided into four regions: The N-terminal RNA-binding domain (RBD; amino acids 1–73), the linker sequence (L; amino acids 74–88), the effector domain (ED; amino acids 89–202), and the C-terminal tail (CTT; amino acids 203-219/230/237). Note that both the L and the CTT can vary in length among different IAV strains, and, although a 237 amino-acids-length NS1 has been represented, IAV NS1 can be 219, 230, or 237 amino acids long. Nuclear localization and export signals (NLS and NES, respectively) are indicated with black boxes at the bottom, including their amino acid locations in the NS1.

The innate immune system is the first line of defense against viral infections, and viruses have to develop efficient countermeasures that allow them to replicate in infected cells and to be propagated to other hosts (Garcia-Sastre A et al., 1998; Kochs et al., 2007; Hale et al., 2008; Nogales et al., 2018b; Nogales et al., 2019b). IAV NS1 is a multifunctional protein and virulence factor, which main role is to counteract or modulate host antiviral interferon (IFN) responses at multiple levels (**Figure 3**) (Garcia-Sastre A et al., 1998; Talon et al., 2000b; Kochs et al., 2007; Hale et al., 2008; Thulasi Raman and Zhou, 2016; Nogales et al., 2018b). Thus, IAV NS1 is required to evade the host innate immune system and to replicate efficiently in IFN-competent systems. NS1 is encoded by the viral segment 8 (or NS) as a primary mRNA transcript (**Figure 3A**). In addition, through an alternative splicing mechanism, a less abundant spliced product encodes for the viral NEP that is essential for IAV replication (Lamb and Lai, 1980; Robb et al., 2010). Depending on the IAV strain (and somehow the targeted host) NS1 is a 219 to 237 amino acid long protein, and four distinct domains have been identified (**Figure 3B**). The N-terminal domain (the first 73 amino acids) contains an RNA-binding domain (RBD) responsible for interacting with double-stranded (ds)RNA, and a nuclear localization signal (NLS) that overlaps with the RBD (Greenspan et al., 1988; Chien et al., 1997; Liu et al., 1997; Wang et al., 1999; Melen et al., 2007; Marc, 2014). Next, a 10–15 amino acids flexible linker (L) domain connects the RBD and effector domain (ED). The ED comprises amino acids 88 to 202 and contains a nuclear export signal (NES) that favors NS1 protein localization at both the nucleus and the cytoplasm during viral infection (Li et al., 1998). The ED is able to interact with multiple host factors, including cellular proteins involved in antiviral responses (Hale et al., 2008; Thulasi Raman and Zhou, 2016; Nogales et al., 2018b). However, many of these interactions can be strain-dependent, and host-adaptation processes could be important for this high variability (Kochs et al., 2007; Steidle et al., 2010; Marc, 2014; Clark et al., 2017; Nogales et al., 2017a; Nogales et al., 2017f; Chauche et al., 2018; Nogales et al., 2018a; Nogales et al., 2021b). The last domain is a C-terminal tail (CTT) of 11–33 amino acids, containing a PDZ-binding motif that is associated with IAV pathogenesis and is not present in all IAV NS1 proteins (Jackson et al., 2008; Hale, 2014). Most IAV strains also have another NLS at the CTT of NS1 (Greenspan et al., 1988) (**Figure 3B**).

**Figure 4:**
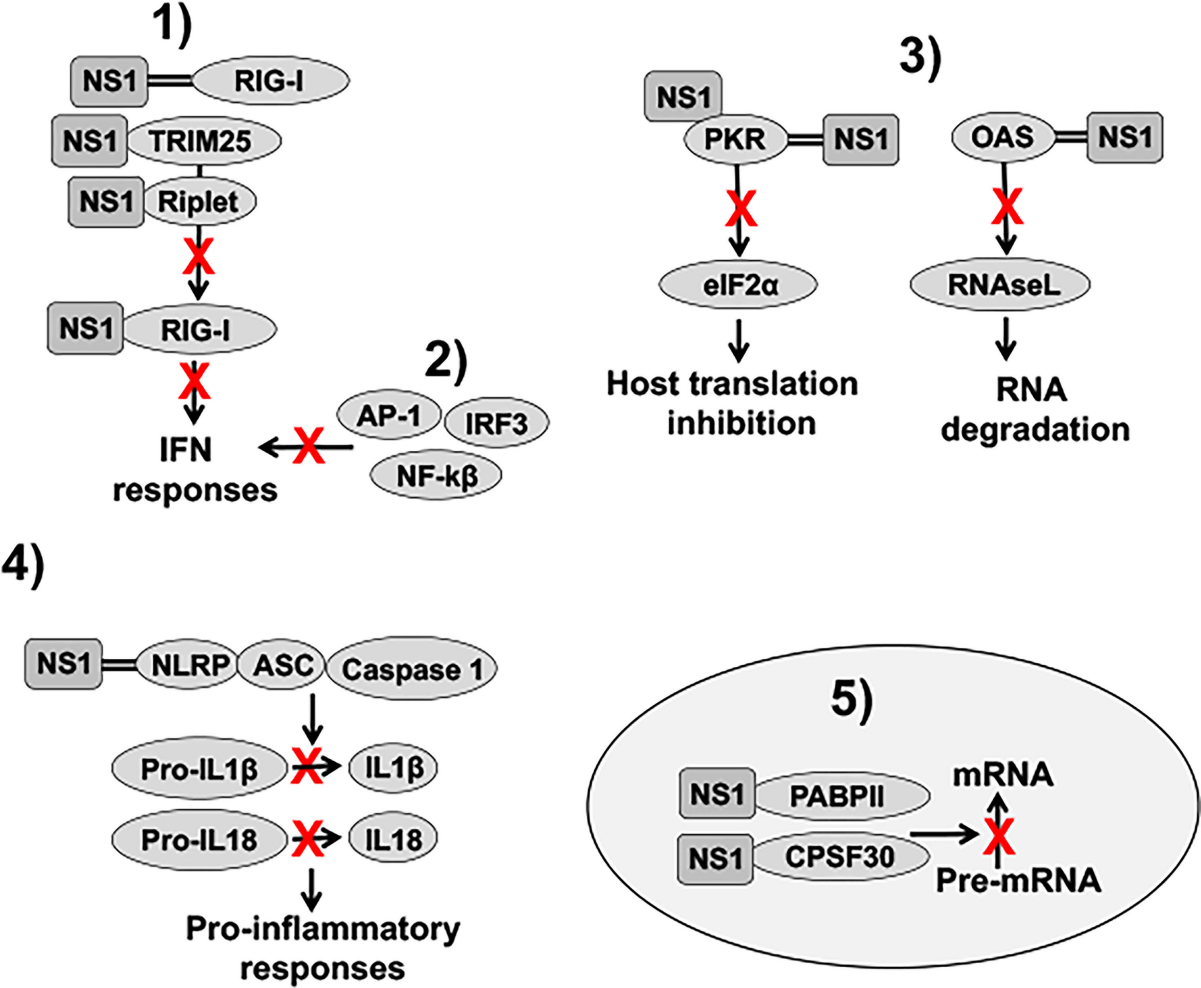
Role of NS1 in counteracting IFN responses. **1**) IAV NS1 decreases RIG-I activation, and therefore, IFN responses, through the sequestration of dsRNA (represented with two parallel lines), or by interaction with RIG-I, TRIM25 or Riplet, resulting in the suppressed ubiquitination and activation of RIG-I. **2**) IAV NS1 inhibits the activation of IRF3, NF-κβ, and AP-1 transcription factors, impairing type I IFN production, and, therefore, the induction of ISGs. (**3**) IAV NS1 directly inhibits the antiviral activities of the ISGs PKR and OAS-RNaseL. NS1 protein binds dsRNA and PKR, leading to decreased PKR activity and impaired host translation inhibition mediated by PKR. IAV NS1 protein also inhibits OAS activation *via* the dsRNA-binding activity of its RBD, therefore, reducing RNA degradation mediated by RNAseL. **4**) IAV NS1 impairs NLRP3 inflammasome activation as well as decreases the cleavage of pro-interleukin (IL)-1β and pro-IL-18 into their mature forms. Upon infection, these cytokines are released from the cell to stimulate inflammatory processes. **5**) Depending on the IAV strain, NS1 proteins can bind to CPSF30. In addition, IAV NS1 binds to PABPII. These interactions of IAV NS1 with CPSF30 and PABPII block the cleavage of immature mRNAs (pre-mRNAs) and the recruitment of the poly(A) polymerase to add the poly(A) tail and function of PABPII to stimulate the synthesis of long poly(A) tails, respectively, leading to host protein shutoff. IFN: interferon; dsRNA: double-stranded RNA; RIG-I: retinoic acid-inducible gene I; TRIM25: tripartite motif containing 25; IRF3: interferon regulatory factor 3; NF-κβ: nuclear factor kappa beta; AP-1: activator protein 1; ISGs: IFN-stimulated genes; PKR: protein kinase R; OAS: 2’,5’-oligoadenylate synthetase; NLRP3: NLR family pyrin domain containing 3; CPSF30: cleavage and polyadenylation specificity factor 30; PABPII: poly(A)-binding protein II.

IAV NS1 inhibits the activation of innate antiviral responses using several molecular mechanisms that have been extensively revised in the literature (**Figure 4**) (Hale et al., 2008; Marc, 2014; Khaperskyy and McCormick, 2015; Nogales et al., 2018b). These include: 1) inhibition of IFN activation through the interaction with retinoic acid-inducible gene I (RIG-I), an intracellular sensor of virus infection (Guo et al., 2007; Mibayashi et al., 2007; Opitz et al., 2007), by sequestering dsRNA during viral infection (Wang et al., 1999), or by interaction with Tripartite Motif Containing 25 (TRIM25) or Riplet, which results in the suppressed ubiquitination and activation of RIG-I (Gack et al., 2009; Rajsbaum et al., 2012; Koliopoulos et al., 2018); 2) inhibition of IFN regulatory factor 3 (IRF3), activator protein 1 (AP-1) and nuclear factor kappa beta (NF-κB) transcription factor activation, including induction of IFN-stimulated genes (ISGs) (Talon et al., 2000a; Wang et al., 2000; Ludwig et al., 2002; Gao et al., 2012); 3) inhibition of specific ISG products, including protein kinase R (PKR) or 2’,5’-oligoadenylate (2-5A) synthetase (OAS)-RNaseL (Hale et al., 2008; Nogales et al., 2018b); 4) inhibition of inflammasome activation by interaction with the NLR family pyrin domain containing 3 **(**NLRP3) (Chung et al., 2015; Moriyama et al., 2016; Park et al., 2018); and 5) inhibition of 3′ end processing and blocking the export of host mRNAs from the nucleus, which leads to the inhibition of cellular gene expression, including IFN, ISG and pro-inflammatory responses by interaction with components of the cellular pre-mRNA processing machinery such as the cleavage and polyadenylation specificity factor 30 (CPSF30) and poly(A)-binding protein II (PABPII) (Chen et al., 1999; Noah et al., 2003) (**Figure 4**). IAV NS1 protein is also involved in viral RNA synthesis and viral replication, translation, and NS1 polymorphisms accumulated over time can be important for host adaptation through multiple protein-protein interactions (Thulasi Raman and Zhou, 2016; Clark et al., 2017; Nogales et al., 2017f; Chauche et al., 2018; Nogales et al., 2018a; Evseev and Magor, 2021). Because the high variability of IAV NS1 functions and interactions with other viral and host factors, the effect of NS1 truncations, or deletion, could, therefore, be different among IAV strains that infected different animal host species.

## NS1 truncated or deficient viruses as LAIV

Vaccines are the most efficient strategy for preventing influenza illness (Jin and Chen, 2014; Martinez-Sobrido et al., 2018; Blanco-Lobo et al., 2019). In addition, because IAV is circulating in multiple mammalian and avian species, vaccines could reduce or prevent the impact of zoonotic events, including potential pandemics, controlling the spread of IAV within these animal reservoirs (Martinez-Sobrido et al., 2020). Moreover, surveillance studies can be key to control the presence of IAV in these host populations and anticipate the emergence of new strains with pandemic potential to humans (Martinez-Sobrido et al., 2020).

**Figure 5:**
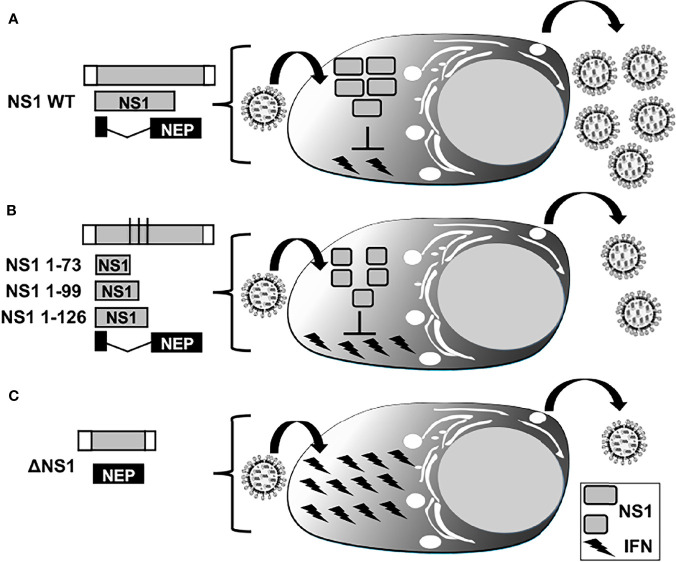
IFN responses in LAIV based on NS1 truncations or complete deletion. Representation of WT **(A)**, truncated **(B)**, or deficient **(C)** NS1 recombinant IAV. WT NS vRNA is represented in gray boxes and NCR located at the 3´and 5´ ends of the NS vRNA are indicated with white boxes. WT NS1 and NEP ORFs are represented as gray and black boxes, respectively. Black lines in panel B represent stop codons. Expression of WT NS1 protein **(A)** results in strong inhibition of IFN induction and, therefore, efficient viral replication. NS1 1–73 (top), 1–99 (middle), or 1–126 (bottom) truncations in the NS1 ORF **(B)** or deletion of the entire NS1 ORF **(C)** result in less efficient inhibition of IFN induction and, thus, reduced viral replication.

Because of IAV NS1’s ability to modulate cellular immune responses and inhibit IFN production (**Figure 4**), multiple vaccine approaches based on the use of modified NS1 proteins as a means for virus attenuation have been developed and assessed (Quinlivan et al., 2005; Solorzano et al., 2005; Richt et al., 2006; Vincent et al., 2007; Wang et al., 2008; Chambers et al., 2009; Steel et al., 2009; Kappes et al., 2012; Choi et al., 2015; Sridhar et al., 2015; Jang et al., 2016; Na et al., 2016; Chen et al., 2017; Hoft et al., 2017; Nogales et al., 2017b; Nogales et al., 2017e; Jang et al., 2018; Nicolodi et al., 2019; Lee et al., 2021; Vandoorn et al., 2022a). NS1 deficient or truncated IAV have been considered as promising LAIV because they replicate poorly in IFN-competent hosts (**Figure 5**), while they are able to induce a strong and protective immune response against WT forms of the virus (Quinlivan et al., 2005; Solorzano et al., 2005; Richt et al., 2006; Vincent et al., 2007; Wang et al., 2008; Chambers et al., 2009; Steel et al., 2009; Kappes et al., 2012; Choi et al., 2015; Sridhar et al., 2015; Jang et al., 2016; Na et al., 2016; Chen et al., 2017; Hoft et al., 2017; Nogales et al., 2017b; Nogales et al., 2017e; Jang et al., 2018; Nicolodi et al., 2019; Lee et al., 2021; Vandoorn et al., 2022a). Importantly, NS1 deficient and/or truncated viruses can grow in appropriated substrates such as IFN-deficient cells (e.g., Vero cells) or systems with an undeveloped IFN system (e.g., 5-6 day-old chicken embryonated eggs) (Garcia-Sastre et al., 1998; Vlecken et al., 2013), required for vaccine production (Bardiya and Bae, 2005; Barrett et al., 2009; Hussain et al., 2010; Perdue et al., 2011; Jin and Chen, 2014). Another advantage of LAIV based on NS1 deficient or truncated viruses is their ability to induct robust both humoral and cellular immune responses (Quinlivan et al., 2005; Solorzano et al., 2005; Richt et al., 2006; Vincent et al., 2007; Wang et al., 2008; Chambers et al., 2009; Steel et al., 2009; Kappes et al., 2012; Choi et al., 2015; Sridhar et al., 2015; Jang et al., 2016; Na et al., 2016; Chen et al., 2017; Hoft et al., 2017; Nogales et al., 2017b; Nogales et al., 2017e; Jang et al., 2018; Nicolodi et al., 2019; Lee et al., 2021; Vandoorn et al., 2022a). While humoral responses are highly important against homologous IAV strains, cellular responses could provide cross-protection against heterologous strains (Nogales et al., 2017e; Korenkov et al., 2018; Rodriguez et al., 2018b; Krammer, 2019; Rodriguez et al., 2019; Smith et al., 2019). Therefore, as broadly protective vaccines are needed, LAIV typically are a better option to protect against heterologous strains than inactivated influenza vaccines (IIV) (Sridhar et al., 2015; Hoft et al., 2017; Nogales et al., 2017e). Recombinant swine (Solorzano et al., 2005; Richt et al., 2006; Vincent et al., 2007; Kappes et al., 2012; Lee et al., 2021; Vandoorn et al., 2022a), equine (Quinlivan et al., 2005; Chambers et al., 2009; Na et al., 2016), canine (Nogales et al., 2017b), and avian (Wang et al., 2008; Steel et al., 2009; Choi et al., 2015; Jang et al., 2016; Chen et al., 2017; Jang et al., 2018; Nicolodi et al., 2019) IAV with different partial truncations or deficient in NS1 have been generated and proposed as potential LAIV in different animal models of infection, including mice (Talon et al., 2000b; Quinlivan et al., 2005; Steel et al., 2009; Pica et al., 2012; Choi et al., 2015; Na et al., 2016; Nogales et al., 2017b), pigs (Richt et al., 2006; Vincent et al., 2007; Kappes et al., 2012; Lee et al., 2021; Vandoorn et al., 2022a), horses (Chambers et al., 2009), and birds (Wang et al., 2008; Steel et al., 2009; Jang et al., 2016; Chen et al., 2017; Jang et al., 2018) (**Table 1**).

**Table 1:** NS1 truncated or deficient viruses used as LAIV.

Host	IAV strain	NS1 modification	Other changes	Animal model	Safety, immunogenicity and protection efficacy (homologous/heterologous or heterosubtypic)	References
Pig	A/Swine/Texas/4199-2/98 H3N2	NS1 1-73, NS1 1-99, NS1 1-126.	NA	Pig	+, +, and (H3N2+/H1N1+). Immunogenicity and protection was evaluated using NS1 1-126.	(Solorzano et al., 2005; Richt et al., 2006; Vincent et al., 2007; Kappes et al., 2012; Vandoorn et al., 2022a)
Pig	6 internal genes from bat/Guatemala/164/2009 H17N10 (Bat09) and the HA and NA from A/Swine/Texas/4199-2/98 H3N2 with Bat09 respective gene packaging signals	NS1 1-128	NS segment +/-swine IL-18.	Pig	+, +, and (Not evaluated/H3N2 +). IL-18 expression did not impact in vaccine efficacy.	(Lee et al., 2021)
Horse	A/equine/Kentucky/5/2002 H3N8	NS1 1-73, NS1 1-99, NS1 1-126.	NA	Mouse and horse	Mouse: Reduce viral replication was shown. Horses: +, +, and (H3N2+/Not evaluated). Protection was evaluated using only NS1 1-126.	(Quinlivan et al., 2005; Chambers et al., 2009)
Dog	A/canine/NY/dog23/2009 H3N8	NS1 1-73, NS1 1-99, NS1 1-126 ΔNS1	NA	Mouse	+, +, and (+/Not evaluated)	(Nogales et al., 2017b)
Poultry	A/Viet Nam/1203/2004 H5N1	NS1 1-73, NS1 1-99, NS1 1-126.	Polybasic cleavage site removed in HA. PB2 containing either K627 or E627 amino acid.	Mouse and chicken	Mouse: +, +, and (+/Not evaluated). Chicken: +, +, and (+/+). Only the virus NS1-99/PB2 E627 was tested in chickens.	(Steel et al., 2009)
Poultry	6 internal genes from PR8 H1N1 and HA and NA from A/WB/Korea/ma81/06 H5N2 and A/CK/Korea/116/03 H9N2, respectively.	NS1 1-73, NS1 1-86, NS1 1-101, NS1 1-122.	NA	Mouse	+, +, and (+/+).	(Choi et al., 2015)
Poultry	A/turkey/Oregon/71 H7N3	NS1 1-91/93 NS1 1-115/125	NA	Chicken	+, +, and (+/+).	(Wang et al., 2008; Jang et al., 2016)
Poultry	A/chicken/Taixing/10/2010 H9N2	NS1 1-73, NS1 1-100, NS1 1-128.	NA	Chicken	+, +, and (+/+). Protection was evaluated using only NS1-128.	(Chen et al., 2017)
Human	IVR-116 (A/New Caledonia/20/1999-like H1N1) and the HA, NA and M segments from A/Viet Nam/1203/2004 H5N1	ΔNS1	NA	Human	+, +, and (Not evaluated).	(Nicolodi et al., 2019)

***NA,** Not available.*

Both B and T cells responses are important for immunity against IAV infection and virus clearance (Blanco-Lobo et al., 2019; Krammer, 2019; Smith et al., 2019; Topham et al., 2021). Dendritic cells (DCs) and macrophages are antigen presenting cells (APCs) playing a key role as mediators between innate and the adaptive immune responses (Akira et al., 2006; Trinchieri and Sher, 2007; Borderia et al., 2008; Monteagudo et al., 2019). Moreover, APCs are able to activate adaptive immune responses against the invading pathogens by triggering T cell differentiation (Cuddapah et al., 2010; Topham et al., 2021; Rattan et al., 2022). Importantly, these cells constantly inspect the lungs for pathogens and they are essential to protect the host against invaders. Previous studies have shown that the induction of a genetic program underlying DCs maturation, migration, and T-cell stimulatory activity is specifically suppressed by IAV NS1 (Fernandez-Sesma et al., 2006). Thus, the role of IAV NS1 in counteracting host responses is not limited to innate immunity evasion but also to inhibition of adaptive immunity *via* modulating the maturation and the capacity of DCs to induce T cell responses. This finding is important because it supports the implementation of LAIV encoding truncated NS1 proteins.

Another advantage of using viruses expressing truncated NS1 proteins is their temperature sensitive (*ts*) phenotype (Falcon et al., 2004; Falcon et al., 2005; Nogales et al., 2017b; Nogales et al., 2019b). It has been shown that NS1 truncated viruses (NS1 1-81 and 1-110) replicated similarly to WT virus in Madin–Darby canine kidney (MDCK) cells at 32°C (Falcon et al., 2004). However, these NS1-truncated viruses showed a *ts* phenotype when replication was evaluated at 39 °C. The molecular basis of the *ts* phenotype of these NS1 mutant viruses was evaluated. At the restrictive temperature, the ratio of replication to transcription activities of vRNPs was altered, showing that NS1 plays a role in virus RNA replication, most likely at the cRNA-to-vRNA step, and that a defect in this function may contribute to the *ts* phenotype observed (Falcon et al., 2004). The *in vitro* results were further confirmed *in vivo* since these NS1 mutant viruses were not detected in the lungs of infected mice at day 3 post-infection, indicating that viruses were also attenuated *in vivo* (Falcon et al., 2005). Notably, mice inoculated with the NS1 truncated viruses were protected against a lethal challenge with WT IAV and specific and robust cellular and humoral immune responses were induced after immunization, supporting the feasibility of using these NS1 truncated IAV as safe, immunogenic and protective LAIV to prevent IAV infection (Falcon et al., 2005). In another study, the variability of NS1 protein from seasonal IAV H3N2 isolated from infected subjects during the 2010/2011 influenza season was analyzed, and amino acid changes in positions 86, 189, and 194 were identified (Nogales et al., 2017c). Interestingly, NS1 mutations D189N and V194I impaired the ability of the NS1 protein to inhibit general gene expression (Nogales et al., 2017c). Moreover, viruses encoding a V194I amino acid change in NS1 displayed a *ts* phenotype, and they were highly attenuated *in vivo* (Nogales et al., 2017c).

An important characteristic to be considered during the development of LAIV is the possibility of the virus in the vaccine to revert to WT or to acquire a virulent phenotype (Zhou et al., 2016). However, this possibility is highly unlikely in the case of NS1 truncated or deficient viruses based on their reduced *in vivo* replication, although additional work will be required to completely discard this possibility. Likewise, the possibility of NS1 truncated or deficient viruses to reassort with IAV strains in the field is also improbable based on their limited *in vivo* replication (Talon et al., 2000b; Quinlivan et al., 2005; Richt et al., 2006; Vincent et al., 2007; Wang et al., 2008; Chambers et al., 2009; Steel et al., 2009; Kappes et al., 2012; Pica et al., 2012; Choi et al., 2015; Jang et al., 2016; Na et al., 2016; Chen et al., 2017; Nogales et al., 2017b; Jang et al., 2018; Lee et al., 2021; Vandoorn et al., 2022a). However, recent studies related with the use of a LAIV encoding an NS1 truncated protein to prevent swine influenza virus has shown that this risk cannot be completely discarded (Mancera Gracia et al., 2020; Sharma et al., 2020), highlighting the importance of IAV surveillance to detect emerging IAV strains with LAIV genes after vaccination campaigns to rule out the potential reassortment between viruses in the vaccine and natural circulating isolates.

### Swine influenza virus (SIV)

SIV is an important pathogen for the swine industry worldwide because pig production has been intensified in the last decades (Salvesen and Whitelaw, 2021). Infection of pigs with SIV can cause respiratory illness, fever, coughing and loss of appetite, and these clinical symptoms contribute to weight loss of animals and thus the reduction of pig industry productivity (Janke, 2013; Salvesen and Whitelaw, 2021). Currently, there are three major subtypes of SIV circulating worldwide in pigs: H1N1, H1N2 and H3N2 (Lewis et al., 2016; Rajao et al., 2018). However, SIV, as other IAV, can evolve rapidly by antigenic shift and antigenic drift  (Parrish et al., 2015; Rajao et al., 2018; Salvesen and Whitelaw, 2021), and thus multiple antigenically distinct SIV strains may be co-circulating in the field. In fact, the picture is further complicated with the existence of different circulating lineages and clades of SIV between countries or regions (Lewis et al., 2016; Henritzi et al., 2020). Therefore, development of effective SIV vaccines is difficult, mainly because the limited cross-protection efficacy against all these SIV subtypes and strains (Brockwell-Staats et al., 2009; Salvesen and Whitelaw, 2021).

Importantly, SIV are considered as potential “mixing vessel” of IAV favoring reassortment between strains of human and animal (mainly avian) origin, when more than one virus infects the same cell (Brockwell-Staats et al., 2009; Garten et al., 2009; Rajao et al., 2018). This characteristic may lead to the generation of potentially zoonotic and/or pandemic influenza strains (Brockwell-Staats et al., 2009; Garten et al., 2009; Rajao et al., 2018; Henritzi et al., 2020). In fact, cross-species transmission of IAV between humans and pigs has been reported (Rajao et al., 2018). In addition, reverse zoonosis from humans to pigs also occur at a high rate (Rajao et al., 2018; Henritzi et al., 2020). IIV are the most widely used prophylactic measure to prevent SIV infections in pigs, and usually they contain a combination of antigenically distinct H1 and H3 subtypes of SIV (Salvesen and Whitelaw, 2021). Although IIV for SIV induce neutralizing antibodies against antigenically similar SIV strains, they offer only partial protection against heterologous strains, similar to the situation seeing in humans (Hoft et al., 2017). On the other hand, LAIV provide a better cross-reactive immunity against antigenically distinct SIV through inducing also robust cell-mediated immune responses (Richt et al., 2006; Vincent et al., 2007; Mancera Gracia et al., 2020; Salvesen and Whitelaw, 2021; Vandoorn et al., 2022a). Moreover, LAIV also induce potent local humoral and mucosal immune responses, contributing to a better protection against IAV infection (Hoft et al., 2017; Salvesen and Whitelaw, 2021). Because there is not universal IAV vaccine technologies that can be used to control SIV infections, it is highly necessary to develop novel and more effective vaccine approaches against currently circulating diverse SIV subtypes and strains, which is also important to reduce the risk of zoonotic transmission to humans (Brockwell-Staats et al., 2009; Rajao et al., 2018; Mancera Gracia et al., 2020). Although several attempts have been developed to produce vaccines for the prevention of SIV infections in pigs, in this review we focus on the implementation of NS1 truncated or deficient viruses for their use as LAIV for the prevention of SIV infections (**Table 1**).

**Figure 6:**
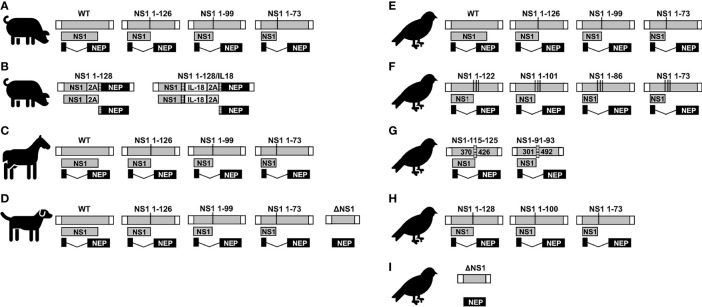
NS1 truncated or deficient IAV as LAIV for SIV (A and B), EIV (C), CIV (D), and AIV **(E–I).** Schematic representation of the WT and modified NS segments for SIV **(A, B)**, EIV **(C)**, CIV **(D)**, and AIV **(E–I)**. NS vRNA is represented in gray boxes and the NCR located at the 3´and 5´ ends of the NS vRNA are indicated with white boxes. NS1 and NEP ORFs are represented as gray and black boxes, respectively. Black lines represent stop codons. In **(B)**, truncated NS1 protein is expressed as a single polyprotein together with 2A autoproteolytic cleavage site and NEP. NEP protein is released from NS1 protein during translation. IL-18 was incorporated between NS1 and NEP proteins *via* GSGG and GSG linkers (striped rectangles), and the 2A autoproteolytic cleavage site. Splice acceptor site was mutated to inhibit splicing. In **(F)**, the NS segments encoded unmodified NEP and truncated NS1 protein products created by adding three serial stop codons comprising amino acids 1-73, 1-86, 1-101, and 1-122 without any nucleotide deletions. In **(G)**, the internal deletion comprising nucleotides 370-426 (NS1-115-125) or 301-492 (NS1-91-93) in NS1 are indicated. The CCT of these NS1 proteins contains the same 115 and 91, respectively, amino acid residues than the WT NS1 but additional different residues (116 to 125 for NS1-115-125; and 92 to 93 in NS1-91/93, respectively) due to a frame shift in the ORF.

Reverse genetics were used to generate recombinant viruses with the goal to study the role of NS1 in the virulence of A/Swine/Texas/4199-2/98 H3N2 (TX/98), an SIV isolate in pigs (Solorzano et al., 2005). For that, authors generated recombinant WT and C-terminally truncated forms of the NS1 protein encoding the first 73, 99, or 126 amino acids, instead of the 219 amino acids of the WT NS1 protein (**Figure 6A**). Results indicated that NS1 mutant viruses displayed a decreased ability to prevent IFN synthesis in pig cells after infection. Moreover, the three NS1 mutant TX/98 viruses were also attenuated in pigs, the natural host, showing reduced viral replication and percentage of lesions in the lungs of infected animals. These results suggest the feasibility of using these NS1 truncated TX/98 viruses as potential LAIV for the prevention of SIV infections in pigs (Solorzano et al., 2005). In fact, in a follow up study, the SIV TX/98 encoding a truncated NS1 protein of 126 amino acids (TX/98 NS1 1-126) was used to vaccinate pigs (Richt et al., 2006). After intranasal vaccination, animals were challenged with WT homologous H3N2 TX/98 or heterosubtypic A/swine/Minnesota/37866/99 H1N1 SIV and sacrificed 5 days later. TX/98 NS1 1-126 completely protected vaccinated pigs against the homologous challenge with H3N2 TX/98 WT. Although macroscopic lung lesions similar to those of the mock-vaccinated H1N1 control pigs were observed in animals challenged with the heterosubtypic SIV H1N1, vaccinated pigs had lower microscopic lung lesions (Richt et al., 2006). Moreover, in this group of vaccinated animals less virus shedding from the respiratory tract was observed, as compared with unvaccinated, H1N1-challenged pigs. Importantly, robust humoral responses against H3N2 TX/98 SIV were induced in vaccinated animals (Richt et al., 2006). As expected, vaccinated animals were seronegative for NS1, allowing the compatibility of this LAIV as a promising DIVA strategy.

In another related later study, TX/98 NS1 1-126 mutant SIV was used to evaluate different routes of vaccination in pigs, as the intranasal immunization has several technical limitations to be applied in the field (Vincent et al., 2007). Authors compared the intramuscular and intranasal routes, showing that the intranasal route induced a strong local (mucosal) immune response. TX/98 NS1 1-126 was shown to provide complete protection efficacy against challenge with a homologous virus when 1 or 2 doses were given intranasally and when 2 doses were given intramuscularly (Vincent et al., 2007). However, only partial protection was induced by intramuscular vaccination with a single dose of TX/98 NS1 1-126. In addition, this LAIV was able to protect against a heterologous homotypic A/SW/CO/23619/99 H3N2 virus and provided partial protection against a heterosubtypic A/SW/IA/00239/2004 rH1N1 virus when administered *via* the intranasal route (2 doses) (Vincent et al., 2007). More recently, the T-cell priming and cross-protective efficacy in weanling piglets after intranasal inoculation with TX/98 NS1 1-126 mutant *versus* WT TX/98 SIV was evaluated (Kappes et al., 2012). Vaccine primed T cells were raised in peripheral blood after inoculation with TX/98 NS1 1-126. As expected, T-cell responses were superior in animals inoculated with the WT virus *in vitro* re-stimulation assays. According to the expression of activation marker CD25, peripheral T-cell recall responses in TX/98 NS1 1-126 infected animals were minimal. Nevertheless, intracellular IFN-γ data at 28 days post-inoculation showed that TX/98 NS1 1-126 was able to induct virus-specific CD4+CD8-, CD4+CD8+, CD4-CD8+, and γδ T cells. Moreover, immunization with TX/98 NS1 1-126 was associated with significantly lower levels of Th1-associated cytokines in infected lungs but still provided partial cross-protection against a challenge with SIV H1N1 (Kappes et al., 2012). These results indicate that NS1 truncated SIV, and specifically TX/98 NS1 1-126, are able to elicit robust cell-mediated cross-protection against antigenically divergent SIV H1N1 strains. In this sense, the efficacy of this SIV LAIV (TX/98 NS1 1-126) against infection with the major North American and European H3N2 SIV lineages was recently studied in another report (Vandoorn et al., 2022a). Results suggest the presence of partial cross-protection against heterologous North American cluster II and IV H3N2 SIV strains (TX/98 NS1 1-126 is based on a cluster I H3N2) (Vandoorn et al., 2022a). TX/98 NS1 1-126 prevented substantial nasal shedding of a North American novel human-like H3N2 SIV, and reduced replication of a European H3N2 SIV. Although this LAIV elicited neutralizing antibodies against homologous virus in serum, no significant cross-reactive antibody titers against the heterologous SIV were observed, suggesting that partial cross-protection relies on cellular and mucosal immune responses against conserved antigens or epitopes of the SIV proteins (Vandoorn et al., 2022a). Since TX/98 NS1 1-126 can offer only partial protection against a broad range of H3N2 SIV strains, it can be still a suitable option for its use in a heterologous prime-boost vaccination strategy. Importantly, in a related study it was examined the pathobiology, type I IFN induction, of TX/98 NS1 1-126 in pigs and compared it with IFN induction in pig kidney-15 (PK-15) cells (Vandoorn et al., 2022b). In PK-15 cells, TX/98 NS1 1-126 induced higher levels of type I IFN than WT TX/98, while virus replication kinetics were similar, although this effect was observed only when cells were infected at high multiplicity of infection. Moreover, nasal excretion from animals intranasally inoculated with the virus TX/98 NS1 1-126 reached titers of up to 4.3 log_10_ 50% tissue culture infective doses (TCID_50_)/mL, although this average titer was 50 times lower than that for WT TX/98. Notably, viral titers of the LAIV in the lower respiratory tract were significantly reduced at 18 to 48 hours but similar to WT TX/98 titers at 72 hours, after intratracheal inoculation. TX/98 NS1 1-126 also caused in general milder clinical signs than WT TX/98 but induced comparable levels of macroscopic and microscopic lung lesions, peak neutrophil infiltration, and peak type I IFN. Thus, authors suggest that although TX/98 NS1 1-126 is partly attenuated in pigs, this could not be linked with higher IFN levels (Vandoorn et al., 2022b).

In 2017, TX/98 NS1 1-126 was the first LAIV licensed in the United States (US) for the prevention of SIV in pigs from 1 day of age. Ingelvac Provenza (Boehringer Ingelheim, St. Joseph, MO, USA) is a bivalent LAIV containing one cluster I H3N2 virus based on TX/98 with the NS1 truncation and one virus containing the same backbone but with the HA and NA derived from a γ2-β-likeH1N1 strain (A/swine/Minnesota/37866/1999), that no longer circulated when the LAIV became available. This vaccine was efficacious in reducing virus nasal shedding after challenge with heterologous strains, either H1N1 or H3N2 (Genzow et al., 2018; Kaiser et al., 2019). However, a phylogenetic analysis of whole genome sequences carried out in the US using samples obtained in 2018 indicated that reassortment strains containing LAIV genes in combination with genes from endemic field strains circulating in US were generated (Mancera Gracia et al., 2020; Sharma et al., 2020), which suggests a substantial degree of LAIV replication. Therefore, this data indicate that reassortment between this SIV LAIV and field strains is possible, although the impact of these reassortment viruses is still unclear and more studies are required. Because the use of this LAIV interfered with routine SIV surveillance in the US, the vaccine was withdrawn from the market in 2020 (https://www.aphis.usda.gov/aphis/ourfocus/animalhealth/veterinary-biologics/product-summaries/vet-label-data/614d8792-aeb1-4837-b04e-50f8c181113f). Notably, different approaches could be used to prevent the risk of reassortment between viruses present in the LAIV and natural circulating isolates (Gao and Palese, 2009; Chen et al., 2020).

Recently, an innovative approach to generate LAIV to prevent SIV infections was reported (Lee et al., 2021). To avoid potential reassortments between viruses present in the LAIV and circulating field strains (Lee et al., 2021), the authors engineered two recombinant chimeric IAV that contained the HA and NA gene open reading frames (ORFs) of the TX/98 SIV and six internal genes from a recently identified bat IAV bat/Guatemala/164/2009 H17N10 (Lee et al., 2021). In addition, the recombinant viruses encoded a C-terminally truncated form of the NS1 protein expressing the first 128 amino acids with or without the swine IL-18 (Bat09:mH3mN2-NS1-128 and Bat09:mH3mN2-NS1-128-IL-18, respectively) (**Figure 6B**) using a 2A autoproteolytic cleavage site strategy to express NS1 and NEP from the same transcript, as was previously described (Nogales et al., 2015; Nogales et al., 2019a). These two new LAIV and an IIV control were tested in pigs against a heterologous KS-91088 H3N2 virus, a reassortant of A/swine/Kansas/10-91088/2010 H3N2 containing the NP, M, and NS genes from the pandemic influenza A/California/04/2009 H1N1 (pH1N1). Compared to the IIV, both LAIV were able to limit nasal virus shedding and reduce lesions and virus replication in lungs. Moreover, LAIV induced greater levels of mucosal IgA responses in the lungs and increased numbers of antigen-specific IFN-γ secreting cells against the challenge virus. Interestingly, authors did not observe differences between both LAIV, suggesting that IL-18 expression did not significantly impact vaccine efficacy, at least, under their experimental conditions (Lee et al., 2021).

### Equine influenza virus (EIV)

EIV is the causative agent of equine influenza, which is an upper respiratory disease characterized by the development of pyrexia, coughing, dyspnea, and nasal discharge (Landolt, 2014; Singh et al., 2018; Oladunni et al., 2021). EIV affects mainly horses, but also other equids and has a severe impact on the equine industry in most parts of the world (Landolt, 2014; Singh et al., 2018; Oladunni et al., 2021). The first EIV isolated in Europe in 1956 was an H7N7 subtype, which spread towards many regions of the world by the early 1960s (Landolt, 2014; Singh et al., 2018; Oladunni et al., 2021). However, this EIV subtype is believed to have disappeared from the equine population (Landolt, 2014; Oladunni et al., 2021). H3N8 EIV was initially isolated in 1963 and rapidly spread causing major outbreaks around the world, which persist today (Landolt, 2014; Singh et al., 2018; Oladunni et al., 2021). The evolution of EIV H3N8 subtype is driven by antigenic drift, and at the end of the 1980s, H3N8 EIV diverged into two antigenically distinct Eurasian and American lineages (Landolt, 2014; Singh et al., 2018; Oladunni et al., 2021). Around 2000, the American lineage evolved into South-American, Kentucky, and Florida sublineages. The Florida sublineage further evolved into two antigenically distinct clades (clade 1 and clade 2) on the basis of the HA sequence, which are presently co-circulating and co-evolving worldwide (Landolt, 2014; Singh et al., 2018; Oladunni et al., 2021). Currently, clade 1 EIV are predominantly found in the US whereas clade 2 EIV are primarily circulating in Europe and Asia (Landolt, 2014; Singh et al., 2018; Oladunni et al., 2021). Prevention and control of H3N8 EIV in the equine population rely on quarantine, hygiene, and vaccination programs to reduce infection and spread between horses (Major, 2011; Paillot et al., 2016). Due to the frequent international transport of horses, the World Organization for Animal Health (OIE, Office International des Epizooties) recommends including representative viruses from both clades in the composition of H3N8 EIV vaccines (Major, 2011; Paillot et al., 2016).

In 2005, three NS1 mutant viruses containing C-terminally truncated NS1 proteins (NS1 1–73, NS1 1–99, and NS1 1–126) in the backbone of A/equine/Kentucky/5/2002 H3N8 were generated using plasmid-based reverse genetics (**Figure 6C**) (Quinlivan et al., 2005). As expected, authors showed that the NS1 truncated EIV were impaired in their ability to inhibit type I IFN production. Moreover, the NS1 truncated viruses replicated at lower levels than a recombinant WT EIV counterpart in embryonated eggs, MDCK cells, or mice, opening the feasibility of using these NS1 truncated EIV as potential LAIV to prevent influenza virus infection in horses. Disagreeing to other findings with human, swine or, more recently, canine IAV (Talon et al., 2000b; Quinlivan et al., 2005; Solorzano et al., 2005; Pica et al., 2012; Nogales et al., 2017b), authors found that in the case of EIV, the length of the NS1 protein did not correlate with the level of viral attenuation, with mutant EIV expressing the shortest NS1 protein (e.g., NS1 1-73) being less attenuated than viruses encoding larger NS1 proteins (e.g., NS1 1–99 and NS1 1–126) (Quinlivan et al., 2005). Authors attributed these unique findings to differences in protein stability and/or degradation (Quinlivan et al., 2005).

In a follow up study in 2009, the authors demonstrated that aerosol or intranasal immunization of horses with EIV NS1 1-126 was safe and able to protect against developing fever and other clinical signs of infection upon challenge with homologous A/equine/Kentucky/5/2002 H3N8 (Chambers et al., 2009). Moreover, horses vaccinated with EIV NS1 1-126 presented reduced quantities of challenge A/equine/Kentucky/5/2002 H3N8 virus compared to mock-vaccinated controls (Chambers et al., 2009), demonstrating the potential of implementing EIV NS1 1-126 as a safe, immunogenic, and protective LAIV against EIV in its natural host, the horse (**Table 1**).

### Canine influenza virus (CIV)

CIV cause a contagious respiratory disease in dogs (2015; Dalziel et al., 2014; Martinez-Sobrido et al., 2020). Currently, three subtypes of CIV H3N8, H3N2, and H1N1 have been identified in dogs. CIV H3N8, originated from the transfer of H3N8 EIV to dogs around 1999 in the US (Crawford et al., 2005). CIV H3N2 is an avian-origin virus identified around 2005 in China (Lee et al., 2016; Voorhees et al., 2017). CIV H1N1 was recently identified in dogs in China (Wang et al., 2019). Whereas CIV H3N8 affects mainly dogs, CIV H3N2 has also been isolated from cats (2015; Crawford et al., 2005; Parrish et al., 2015; Martinez-Sobrido et al., 2020). Dogs are the most popular companion animal in the world and humans are closely exposed to pathogens affecting dogs. Because that, and the ability of IAV to infect multiple species and cause pandemics, CIV could be an important health concern for humans or other mammalians (Wille and Holmes, 2019; Martinez-Sobrido et al., 2020). Moreover, natural and experimental infections of dogs with human viruses have been reported (Dundon et al., 2010). Thus, reassortant viruses between canine and human IAV could result in the emergence of new viruses with novel properties, including the ability to infect humans. The zoonotic risk potential of CIV highlights the importance of monitoring and controlling CIV infections and spread in dogs, not only for canine health, but also for human well-being. Recently, members of the National Institutes of Allergy and Infectious Diseases (NIAID) Centers of Excellence for Influenza Research and Surveillance (CEIRS) network collaborated to address the public health risk of emerging IAV and their ability to respond to a potential IAV pandemic, using CIV H3N2 as an example (Martinez-Sobrido et al., 2020). The network performed studies specifically addressing the criteria described in the public health algorithms developed by the Centers for Disease Control and Prevention (CDC) and the World Health Organization (WHO) to estimate the potential risk to human health and of pandemic emergence. Data indicated that CIV H3N2 pose a low risk to humans, with younger people representing the highest population at risk (Martinez-Sobrido et al., 2020).

Currently, only IIV are commercially available to prevent infections of dogs with H3N8 and H3N2 CIV subtypes. To date, IIV or LAIV are not yet available for the prevention of CIV H1N1. In addition, the efficacy of these CIV IIV to protect against H3N8 and H3N2 subtypes is not optimal and improved vaccines are needed (Nogales et al., 2017b; Nogales et al., 2017e; Rodriguez et al., 2017a; Rodriguez et al., 2017b). Because usually LAIV induce better immunogenicity and protection efficacy than IIV, several attempts have been evaluated to generate more efficient monovalent or bivalent LAIV for the prevention of CIV infections (Nogales et al., 2017b; Nogales et al., 2017e; Rodriguez et al., 2017a; Rodriguez et al., 2017b). Using plasmid-based reverse genetics approaches, recombinant A/canine/NY/dog23/2009 H3N8 viruses containing a full-length (WT), truncations (NS1 1-73, NS1 1-99, and NS1 1-126) or a deletion (ΔNS1) of the NS1 protein were generated and tested as potential LAIV to prevent CIV infections (**Figure 6D**) (**Table 1**) (Nogales et al., 2017b). Results demonstrate that all NS1 mutant viruses, both NS1 truncated or deficient, were attenuated in a mouse model of infection, but able to confer complete protection against challenge with WT CIV H3N8 after a single intranasal immunization. Importantly, immunogenicity and protection efficacy was better than that of a commercial H3N8 CIV IIV (Nogales et al., 2017b). The viruses containing truncated versions of NS1 (CIV NS1 1-126, NS1 1-99, and NS1 1-73) displayed comparable replication kinetics in MDCK cells than those of WT H3N8 CIV at both 37°C and 33°C. However, ΔNS1 H3N8 CIV displayed a *ts* phenotype and viral replication was affected at 37°C but not at 33°C (Nogales et al., 2017b), as previously described for other NS1 deficient IAV (Falcon et al., 2005). The fact that NS1 truncated H3N8 CIVs replicated efficiently in MDCK cells addresses the concern of vaccine production and commercialization for their implementation as LAIV. Notable, authors also assessed viral replication *ex vivo* using cultured canine tracheal explants and showed reduced levels of viral replication of the NS1 truncated or deficient viruses compared to WT CIV (Nogales et al., 2017b).

### Avian influenza virus (AIV)

AIV infect a number of different avian host species and are classified according to the OIE as low or highly pathogenic AIV (LPAIV and HPAIV, respectively) based on its pathogenicity in domestic chickens. Wild waterbirds are the natural reservoir of LPAIV and from them LPAIV can be transmitted to domestic birds, other wild or domestic animals, and humans (Parrish et al., 2015; Sutton, 2018; Wille and Holmes, 2019; Wang et al., 2021). LPAIV strains can cause mild to severe disease in poultry, and they are associated with mild clinical signs in broilers and reduction in egg production in layers. In addition, LPAIV can promote secondary infections causing an increase in mortality (Mo et al., 1997; Alexander, 2000). LPAIV of the H5 and H7 subtype can evolve into HPAIV upon introduction into poultry, causing systemic and fatal infections with high mortality rates in poultry (Sutton, 2018; Verhagen et al., 2021). HPAIV strains are highly contagious and can be transmitted from poultry to wild birds, in which the viruses can circulate asymptomatically, or cause severe disease and mortality (Mo et al., 1997; Alexander, 2000; Sutton, 2018; Verhagen et al., 2021). An important component of IAV evolution and epidemiology occurs at the wild–domestic interface (Parrish et al., 2015; Short et al., 2015; Wille and Holmes, 2019). Both LPAIV and mainly HPAIV have an enormous economic impact in the poultry industry that have suffered colossal damages due to repeated outbreaks of AIV. In addition, AIV also represent a risk to human health since avian-origin IAV have been key in the last four IAV pandemics (1918, 1957, 1968, and 2009) and they have also been the source of novel IAV strains in other mammalian hosts, including EIV, SIV and CIV (Parrish et al., 2015; Sutton, 2018; Taubenberger and Morens, 2019; Wille and Holmes, 2019; Wang et al., 2021). Disturbingly, HPAIV outbreaks in poultry and wild birds are no longer an occasional phenomenon in the world (Sutton, 2018; Verhagen et al., 2021; Wang et al., 2021). In addition, outbreaks of novel H6, H7, H9, and H10 AIV have been identified in poultry, including zoonotic infections of humans (Arzey et al., 2012; Wang et al., 2021). Fortunately, the absence of sustained human-to-human transmission has limited the impact of AIV in the human population.

The first HPAIV H5N1 was detected in 1996 in geese in China and in 1997 the first human case of an H5N1 HPAIV was reported in a three-year-old boy in Hong Kong, China (Xu et al., 1999; Chan, 2002). Since then, H5 viruses continue to spread, posing a major challenge to both animal and human health. Due to the high mortality associated with HPAIV H5N1 in poultry, more effective vaccines are urgently needed. Using reverse genetics, several new LAIV based on recombinant influenza A/Viet Nam/1203/2004 H5N1 were generated (Steel et al., 2009). Each virus was attenuated through expression of an HA protein in which the polybasic cleavage site had been removed. In addition, viruses encoded a full-length or C-terminally truncated 1-73, 1-99, or 1-126 NS1 protein (**Figure 6E**). Viruses were generated with PB2 containing either K627 or E627 because it was expected that AIV containing E627 would be attenuated in mammalian hosts, and therefore adding an increasing layer of safety for the vaccine to be used in humans (Steel et al., 2009). Although all recombinant H5N1 NS1 mutant viruses replicated at high titers in embryonated chicken eggs, they grew poorly in human A549 cells as compared to a recombinant virus expressing the entire NS1 protein, either in the backbone of K627 or E627 PB2 viral segment (Steel et al., 2009). As expected, high levels of type I IFN were induced in A549 cells infected with NS1 truncated viruses compared to those expressing the full-length WT NS1 protein. *In vivo*, H5N1 NS1 mutant viruses were highly attenuated in mice and intranasal immunization with a single dose of H5N1 NS1 truncated viruses conferred complete protection against an otherwise lethal challenge with a recombinant A/Puerto Rico/8/34 H1N1 (PR8) expressing H5N1 HA and NA glycoproteins (Steel et al., 2009). Based on these initial results, the authors selected the H5N1 NS1 1-99 containing PB2 E627 for further testing in chickens. Interestingly, a single vaccination dose of this AIV LAIV completely protected chickens against an homologous lethal challenge with A/Viet Nam/1203/2004 H5N1 and provided a high level of protection from a heterologous virus, A/egret/Egypt/01/06 H5N1 (Steel et al., 2009). Altogether, these results indicate that LAIV for the prevention of H5N1 AIV can be generated through the introduction of mutations in the viral HA, NS1, and PB2 proteins to prevent HPAIV infections. More importantly, a similar approach could be used for the development of LAIV for the prevention of other AIV, including HPAIV.

In another study, a different strategy to develop a dual LAIV against H5N1 and H9N2 AIV by modifying the NS1 gene was used (Choi et al., 2015). Using the backbone of PR8 expressing truncated NS1 proteins (NS1 1-73, NS1 1-86, NS1 1-101, and NS1 1-122) authors generated viruses containing the HA from A/WB/Korea/ma81/2006 H5N2 (WB/ma81/H5N2) and the NA segment from A/CK/Korea/116/2003 H9N2 (CK/116/H9N2) (**Figure 6F**). H5N2/NS1 1-86 and H5N2/NS1 1-101 were highly attenuated compared to H5N2 expressing the full-length or other remaining NS1 proteins in a mouse model. Notably, intranasal immunization with a single dose of these recombinant H5N2/NS1 1-86 and H5N2/NS1 1-101 viruses completely protected mice from an otherwise lethal challenge with the homologous A/WB/Korea/ma81/06 H5N2 or heterologous A/EM/Korea/W149/06 H5N1, and a heterosubtypic mouse-adapted H9N2 AIV (Choi et al., 2015). Therefore, these data suggest that H5N2/NS1 1-86 and H5N2/NS1 1-101 mutant viruses could be used as LAIV candidates for the prevention of H5N1 influenza infections in mammalian hosts.

In 2016 the association between type I IFN response and protective efficacy of viruses encoding truncated NS1 proteins was analyzed (Jang et al., 2016). This approach could represent an excellent option for the development of vaccine candidates for the prevention of AIV infections in chickens based on their attenuated, immunogenic, and protective phenotype (Jang et al., 2016). In this study, authors analyzed the relationship between induction of type I IFN and ISGs responses *in vivo* and the immunogenicity and protective efficacy of LAIV based on truncations in the NS1, showing that antibody induction and protective efficacy correlates well with upregulation of ISGs. Further, through oral administration of recombinant chicken IFNα in drinking water, authors showed that IFNα can promote rapid induction of adaptive immune responses and protective efficacy of influenza vaccines in chickens (Jang et al., 2016). For this study authors used two NS1 truncated viruses based on the LPAIV A/turkey/Oregon/1971 H7N3, where one mutant expressing the first 91 amino acids of NS1 (pc4-LAIV) was more efficacious than the other expressing the first 115 amino acids of NS1 (pc2-LAIV) in protecting chickens against heterologous A/chicken/NJ/150383-7/02 H7N2 challenge virus (Wang et al., 2008) (**Figure 6G**). In another related study using the mutant NS1 virus pc4-LAIV (NS1 1-91/93) (**Figure 6G**), authors compared the performance of this virus and an IIV in young chickens vaccinated at 1 day of age (Jang et al., 2018). A single dose of pc4-LAIV induced stronger innate and mucosal responses, and protected young immunologically immature chickens, than a single dose of the IIV. Moreover, when 1-day-old animals were intranasally primed with the LAIV and subcutaneously boosted with IIV three weeks later, they showed a rapid, robust, and highly cross-reactive serum antibody response and a high level of mucosal antibody response. These experiments highlight the importance of testing combinations of different vaccine approaches to optimize the protection efficacy or to obtain cross-protection against multiple AIV strains.

In another study, three NS1 truncated mutant viruses of a LPAIV H9N2 virus A/chicken/Taixing/10/2010 (rTX-NS1 1-73, rTX-NS1 1-100, and rTX-NS1 1-128) were rescued (**Figure 6H**) (Chen et al., 2017). All viruses replicated efficiently in embryonated chicken eggs and MDCK cells, which is an important feature for vaccine production. Importantly, all viruses were attenuated in chickens, although the mutant rTX-NS1 1-128 exhibited the most attenuated phenotype and lost transmissibility. The rTX-NS1 1-128 mutant virus also protected vaccinated chickens against homologous (A/chicken/Taixing/10/2010 H9N2) and heterologous (A/chicken/Shanghai/F/98 H9N2) AIV challenge and induced high levels of specific IgA and IgG antibody responses, suggesting that rTX-NS1 1-128 represents an excellent LAIV candidate against H9N2 viruses (Chen et al., 2017).

Although the goal of this review is to describe veterinary LAIV based in NS1 truncated or deficient viruses, because the zoonotic potential of AIV, vaccines against HPAIV could be highly important also for humans if AIV acquires the ability to efficiently transmit among humans (Verhagen et al., 2021; Wang et al., 2021). In this regard, the safety and immunogenicity of a H5N1 virus where the NS1 protein was deleted (H5N1 ΔNS1) was assessed (**Figure 6I**) (Nicolodi et al., 2019). For that, authors generated a recombinant virus containing the 5 internal genes from IVR-116 (A/New Caledonia/20/1999-like H1N1), including the NS1 deletion in the NS segment, and the HA, NA, and M segments from HPAIV A/Viet Nam/1203/2004 H5N1. Authors conducted a phase 1 study in healthy male and female adult participants who received two intranasal immunizations of the H5N1 ΔNS1 LAIV at 6.8 log_10_ TCID_50_/subject, 7.5 log_10_ TCID_50_/subject, or placebo. Results indicated that H5N1 ΔNS1 LAIV was safe and only symptoms associated with mild influenza infections were observed in some vaccinated subjects. Notably, H5N1 ΔNS1 LAIV was able to induce significant specific serum antibody titers even after a single immunization dose (ClinicalTrials.gov identifier NCT03745274). Importantly, the recombinant H5N1 ΔNS1 LAIV was not re-isolated in participants after one immunization (Nicolodi et al., 2019), suggesting that the generation of new reassortants containing genes from seasonal viruses is unlikely.

### NS1 and DIVA vaccines

One of the main concerns with the use of LAIV in animals is to differentiate antibodies produced due to vaccination from antibodies produced in response to natural virus infection (Pasick, 2004; Hasan et al., 2016). In order to overcome this issue and Differentiate Infected from Vaccinated Animals (DIVA), several approaches have been implemented, such as including sentinel non-vaccinated animals, the use of IIV or subunit vaccines, heterologous neuraminidase vaccines, chimeric HA epitope marked vaccines, and developing serological tests against viral proteins which allow to differentiate infected from vaccinated animals among others (Ozaki et al., 2001; Tumpey et al., 2005; Avellaneda et al., 2010; Brahmakshatriya et al., 2010; Wang et al., 2011; Hasan et al., 2016; Sun et al., 2021). The DIVA strategy is not a new concept since it has been previously used for other pathogens (Calvo-Pinilla et al., 2020; Utrilla-Trigo et al., 2022), including the eradication of an infectious disease from a region or country (Pasick, 2004). In addition, DIVA strategies are useful for global trade (e.g., import or export animals and animal products free of a particular disease agent), reducing the risk of introducing new disease to a naïve animal population.

**Figure 7:**
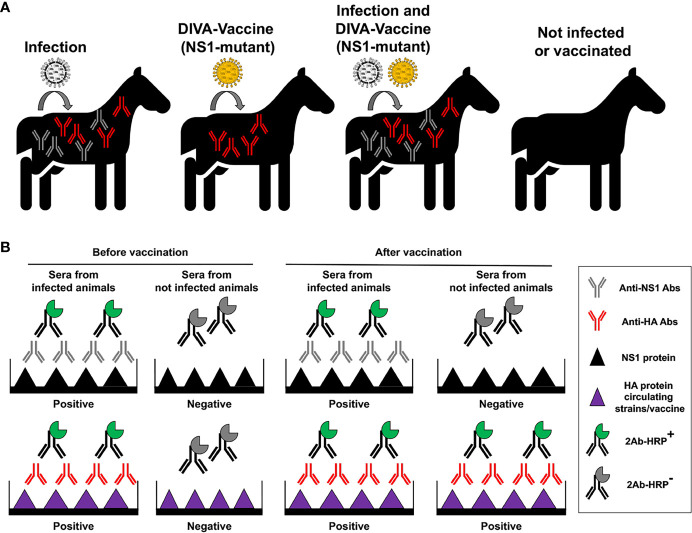
Principle of vaccination with NS1 truncated or deficient viruses as DIVA LAIV. **(A)** Specific antibodies (Abs) against IAV NS1 protein will be produced in infected animals, but not in animals vaccinated with NS1 truncated or deficient IAV (DIVA vaccine). However, specific antibodies against viral HA from circulating strains or the vaccine will be induced in infected and/or vaccinated animals, respectively. **(B)** Development of a DIVA serological test. Abs against IAV NS1 induced by natural infection can be identified by proper serological tests. In the figure an ELISA is used to differentiate sera from infected (containing antibodies against both NS1 and HA proteins) and not infected animals, before vaccination. Wells are coated using recombinant NS1 or HA IAV proteins. Then, the same serological test is carried out after vaccination to differentiate animals that has been infected from animals that have been not infected. Samples from infected animals will be positive for HA and NS1 in the ELISA tests, while samples from vaccinated and not infected animals will be positive only for HA in the ELISA tests.

NS1 is a highly expressed and one of the most abundant proteins produced during IAV infection and, therefore, easily detectable in infected cells (Ozaki et al., 2001), and it has been suggested to be packaged into infective viral particles in small amounts (Hutchinson et al., 2014). Moreover, antibodies against NS1 have been observed in infected animals (Birch-Machin et al., 1997; Ozaki et al., 2001; Tumpey et al., 2005; Dundon et al., 2007; Avellaneda et al., 2010; Brahmakshatriya et al., 2010; Wang et al., 2011; Hasan et al., 2016). These characteristics allow the development of a diagnostic DIVA-antigen approach based on the identification of antibodies against NS1 to differentiate naturally infected animals from those vaccinated with LAIV based on truncated or deficient NS1 mutant viruses (**Figure 7**). Moreover, diagnostic DIVA-NS1 approaches would be useful also for the implementation of other immunization strategies, including IIV. Several studies have showed that this DIVA-NS1 approaches could be used in poultry and that vaccinated/not-infected or vaccinated/infected animals can be differentiated based on the NS1 antibody response (Birch-Machin et al., 1997; Ozaki et al., 2001; Tumpey et al., 2005; Dundon et al., 2007; Avellaneda et al., 2010; Brahmakshatriya et al., 2010; Wang et al., 2011; Hasan et al., 2016). However, the NS1 DIVA strategy has also some limitations including the inconsistent antibody response to the NS1 protein after natural infection, the sometimes poor immune response to NS1 protein in vaccinated individuals, and NS1 protein variability, among others (Birch-Machin et al., 1997; Ozaki et al., 2001; Tumpey et al., 2005; Dundon et al., 2007; Avellaneda et al., 2010; Brahmakshatriya et al., 2010; Wang et al., 2011; Hasan et al., 2016; Evseev and Magor, 2021). Therefore, it will important to develop improved tests to establish highly sensitive, reproducible and effective NS1-based DIVA approaches (Avellaneda et al., 2010; Hasan et al., 2016). Ideally, DIVA tools needs to be highly sensitive, effective in distinguish vaccinate from infected animals, cost effective, and suitable for mass screening. In this sense, ELISA–based diagnostic tests (**Figure 7**) might be an interesting assay to develop, as this can help in scoring a highly sensitive and specific DIVA test. Importantly, novel DIVA assays could take advantage of different strategies such as the combination of NS1-based DIVA approaches with LAIV containing NS1 mutant genes together with heterologous NA, or the identification of other suitable DIVA antigens that could be included in next generation DIVA assays.

## LAIV based on codon-deoptimization (CD) of NS1

**Figure 8:**
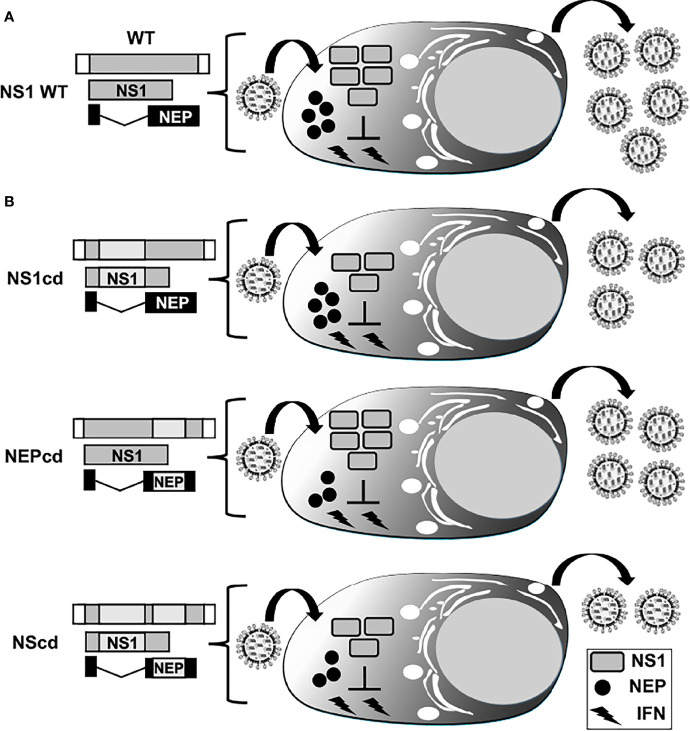
Codon deoptimization of NS segment for the generation of LAIV. Schematic representation of WT **(A)** and codon deoptimized, cd **(B)** viral NS segments. NS vRNA is represented in gray boxes and the NCR located at the 3´and 5´ ends of the NS vRNA are indicated with white boxes. WT NS1 and NEP ORFs are represented as gray and black boxes, respectively. The cd region is represented with light gray boxes for viruses encoding cd NS1 (NS1cd, top), NEP (NEPcd, middle) or both NS1 and NEP (NScd, bottom) proteins. After infection with an IAV encoding a WT NS segment, expression of full-length NS1 results in inhibition of IFN induction, allowing efficient viral replication **(A)**. Infection with IAV NS1cd (top, **(B)** results in reduced NS1 protein expression and inefficient inhibition of IFN responses, resulting in reduced viral replication. Infection with IAV NEPcd (middle, **(B)** results in lower expression of NEP, affecting viral replication. Infection with IAV NScd (bottom, **(B)** results in the virus showing higher attenuation, correlating with the amount of codon changes introduced in both NS1 and NEP in the modified NS viral segment.

Viruses need the translation machinery of infected cells to synthesize their proteins for the formation of virus progeny (Boivin et al., 2010; Thulasi Raman and Zhou, 2016; Rodriguez et al., 2018a; Long et al., 2019). The degeneracy of the genetic code allows for most of the amino acids to be encoded by more than one synonymous codon (Xu et al., 2004). However, most viruses have evolved to modify their codon usage according to the host they infect (Hershberg and Petrov, 2008; Wong et al., 2010; Plotkin and Kudla, 2011; Baker et al., 2015). New biotechnological advances have allowed the generation of recombinant IAV containing genes with deoptimized codons (Nogales et al., 2014; Baker et al., 2015), which could be used as potential LAIV. In fact, we have demonstrated that recombinant IAV containing a codon-deoptimized NS1 protein (NS1cd), alone or in combination with a codon-deoptimized NEP sequence (NEPcd), were attenuated in mice and able to provide, upon a single immunization dose, protection against a lethal challenge with a homologous PR8 or heterologous X31 (a recombinant virus containing the HA and NA segments of A/Hong Kong/1/1968 H3N2 in the background of PR8) IAV, demonstrating the feasibility of implementing this CD-based approach for the development of safe, immunogenic, stable, and protective LAIV for the prevention of IAV infections (Nogales et al., 2014; Baker et al., 2015) (**Figure 8**). Furthermore, LAIV based on CD viruses displayed similar viral replication kinetics to WT virus in MDCK cells, important for vaccine manufacturing and production. Moreover, these additional studies demonstrate that IAV NS1 can be targeted using different approaches for the development of LAIV to protect and prevent IAV infections and spread.

## Conclusions and future directions

Usually, LAIV are able to induce broader and longer-lasting protection than IIV based on their ability to induce induction of robust mucosal humoral immunity and cross-reactive cellular immune responses (Sridhar et al., 2015; Hoft et al., 2017; Nogales et al., 2017e; Korenkov et al., 2018; Rodriguez et al., 2018b; Krammer, 2019; Rodriguez et al., 2019; Smith et al., 2019). Therefore, several efforts have been pursued to generate safer and more effective LAIV approaches. Among them, NS1 mutant IAV have been shown to represent an excellent option for their implementation as safe, immunogenic, stable, and protective LAIV to prevent infection of multiple IAV in different animal species (Talon et al., 2000b; Quinlivan et al., 2005; Solorzano et al., 2005; Richt et al., 2006; Vincent et al., 2007; Wang et al., 2008; Chambers et al., 2009; Steel et al., 2009; Kappes et al., 2012; Pica et al., 2012; Choi et al., 2015; Jang et al., 2016; Na et al., 2016; Chen et al., 2017; Nogales et al., 2017b; Jang et al., 2018; Nicolodi et al., 2019; Lee et al., 2021; Vandoorn et al., 2022a), including humans (**Table 1**). The implementation of reverse genetics methods have been key for the development of novel LAIV approaches to prevent and control IAV infections in different species (Nogales et al., 2016a; Nogales and Martinez-Sobrido, 2016; Blanco-Lobo et al., 2019). Importantly, all the vaccination strategies have advantages and disadvantages that need to be considered carefully. Some of the aspects to be evaluated include the safety, immunogenicity, protection efficacy against different IAV strains, LAIV stability, and the cost of vaccine manufacture, among others.

Attenuation of NS1 mutant truncated or deficient IAV is mainly due to their limited capability to counteract host type I IFN responses, which also increases their immunogenic properties. However, it has been shown that NS1 truncated or deficient IAV can also display a *ts* phenotype, which can further contribute to their attenuation and their ability to induce strong mucosal humoral immunity (Falcon et al., 2005; Nogales et al., 2017b; Nogales et al., 2017c). Importantly, these LAIV have demonstrated efficacy in several avian and mammalian hosts against many IAV strains (Talon et al., 2000b; Quinlivan et al., 2005; Solorzano et al., 2005; Richt et al., 2006; Vincent et al., 2007; Wang et al., 2008; Chambers et al., 2009; Steel et al., 2009; Kappes et al., 2012; Pica et al., 2012; Choi et al., 2015; Jang et al., 2016; Na et al., 2016; Chen et al., 2017; Nogales et al., 2017b; Jang et al., 2018; Nicolodi et al., 2019; Lee et al., 2021; Vandoorn et al., 2022a).

Moreover, the safety and protective immunogenicity of these LAIV can be modulated by introducing different truncations, or deletions, in the viral NS1 protein (Falcon et al., 2005; Quinlivan et al., 2005; Steel et al., 2009; Chen et al., 2017; Nogales et al., 2017b). Nevertheless, more field trials will be required before implementing some of these LAIV approaches, and to determine the reduction of disease symptoms or viral shedding in a real scenario. Also, it will be important to evaluate the possibility of reversion of these LAIV to a virulent phenotype through mutation in other regions of the viral genome or reassortment in the field with naturally circulating IAV strains, as recently described for SIV (Mancera Gracia et al., 2020; Sharma et al., 2020). Likewise, it is important to highlight that LAIV based on NS1 mutants will not overcome the necessity to develop universal vaccines to avoid the consequences of IAV variability, although a better cross-protection will be obtained by using LAIV, as compared to IIV vaccines, when there is an antigenic mismatch between the viruses included in the vaccine and circulating strains (Sridhar et al., 2015; Hoft et al., 2017; Nogales et al., 2017e; Korenkov et al., 2018; Rodriguez et al., 2018b; Krammer, 2019; Rodriguez et al., 2019; Smith et al., 2019). Finally, IAV NS1 deficient or truncated based LAIV-based approaches are compatible with DIVA strategy, which is important for animal immunization to control the dissemination of pathogens (Ozaki et al., 2001; Pasick, 2004; Tumpey et al., 2005; Avellaneda et al., 2010; Brahmakshatriya et al., 2010; Wang et al., 2011; Hasan et al., 2016; Calvo-Pinilla et al., 2020; Utrilla-Trigo et al., 2022). In conclusion, although NS1 truncated or deficient IAV represent an exceptional approach for their implementation as safe, immunogenic, stable, and protective LAIV, there is still a long way to go for their implementation for the prevention of IAV infections. Moreover, LAIV based on NS1 mutant IAV could be combined with other vaccine approaches, including the use of IIV, to optimize the level of cross-protection against multiple IAV strains (Sridhar et al., 2015; Hoft et al., 2017; Jang et al., 2018; Korenkov et al., 2018).

## Author contributions

AN, MD, and LM-S wrote and edited the article. All authors contributed to the article and approved the submitted version.

## Funding

Research on influenza in our laboratories is partially supported by grants W81XWH-18-1-0460-PRMRP-DA from the Department of Defense (DoD) Peer Reviewed Medical Research Program (PRMRP), NIH R01 AI145332-01 and R01AI141607, and by the CEIRR (Center for Research on Influenza Pathogenesis and Transmission), a NIAID funded Center of Excellence for Influenza Research and Response (CEIRR, contract # 75N93021C00014) to LM-S, and by grant RTI-2018-094213-A-I00 from the Spanish Ministry of Science, Innovation, and Universities to MD. This research was partially funded by a “Ramon y Cajal” Incorporation grant (RYC-2017) from the Spanish Ministry of Science, Innovation and Universities to AN.

## Conflict of interest

LM-S and AN have patented LAIV for the prevention of canine and equine IAV.

The authors declare that the research was conducted in the absence of any commercial or financial relationships that could be construed as a potential conflict of interest.

## Publisher’s note

All claims expressed in this article are solely those of the authors and do not necessarily represent those of their affiliated organizations, or those of the publisher, the editors and the reviewers. Any product that may be evaluated in this article, or claim that may be made by its manufacturer, is not guaranteed or endorsed by the publisher.
